# Cancer and the emotions in 18th-century literature

**DOI:** 10.1136/medhum-2018-011639

**Published:** 2019-11-06

**Authors:** Noelle Gallagher

**Affiliations:** English, University of Manchester, Manchester M13 9PL, UK

**Keywords:** literature and medicine, eighteenth-century studies, poetry and prose, British literature

## Abstract

This essay argues that the emotional rhetoric of today’s breast cancer discourse—with its emphasis on stoicism and ‘positive thinking’ in the cancer patient, and its use of sympathetic feeling to encourage charitable giving—has its roots in the long 18th century. While cancer had long been connected with the emotions, 18th-century literature saw it associated with both ‘positive’ and ‘negative’ feelings, and metaphors describing jealousy, love and other sentiments as ‘like a cancer’ were used to highlight the danger of allowing feelings—even benevolent or pleasurable feelings—to flourish unchecked. As the century wore on, breast cancer in particular became an important literary device for exploring the dangers of feeling in women, with writers of both moralising treatises and sentimental novels connecting the growth or development of cancer with the indulgence of feeling, and portraying emotional self-control as the only possible form of resistance against the disease. If, as Barbara Ehrenreich suggests, today’s discourse of ‘positive thinking’ has been mobilised to make patients with breast cancer more accepting of their diagnosis and more cooperative with punitive treatment regimens, then 18th-century fictional exhortations to stay cheerful served similarly conservative political and economic purposes, encouraging continued female submission to male prerogatives inside and outside the household.

In her scathing attack on the cult of positive thinking in modern America, *Smile or Die*, Barbara Ehrenreich declaims against ‘the cheerfulness of breast cancer culture’, with its ‘ultrafeminine’ and ‘infantilizing’ merchandise, its perverse insistence on the ‘redemptive powers of the disease’, and its medically questionable assertions that a positive outlook is ‘essential to recovery’.1 ‘Let me be hacked to death by a madman’, Ehrenreich recalls thinking in a mammography changing booth decorated with pink ribbons, inspirational poetry and breast cancer teddy bears, ‘anything but suffocation by the pink sticky sentiment embodied in that bear and oozing from the walls of the changing room. I didn’t mind dying, but the idea that I should do so while clutching a teddy and with a sweet little smile on my face—well, no amount of philosophy had prepared me for that’.2

While Ehrenreich contrasts the burgeoning public discourse about breast cancer against the attitudes of 40 years ago, when ‘breast cancer was a dread secret, endured in silence and euphemized in obituaries as a ‘long illness’’, the discourse of sentimentality in relation to breast cancer actually has a surprisingly lengthy history.3 In the pages that follow, I want to suggest that the ‘pink sticky sentiment’ Ehrenreich identifies goes back at least as far as the 18th century. While cancer had long been associated with the emotions, the 1700s saw it connected with both positive and negative feelings: it infiltrated literary portraits of maternal kindness and romantic love, as well as warnings against grief, jealousy or pride. In both cases, responsibility for the ‘cancerous’ overgrowth of emotion was located with the victim, as the metaphor was used to warn of the danger of allowing feelings—even good or pleasant feelings—to flourish unchecked.

As the century wore on, breast cancer in particular became a popular plot device within literature designed to inculcate virtue or inspire sympathy, with moralising writers emphasising what Ehrenreich calls the ‘redemptive powers of the disease’, while sentimental writers exploited the piteous tableau of the breast cancer victim dying ‘with a sweet little smile on her face’. Just as many of these texts associated the development or growth of cancer with the indulgence of intense emotion, so they portrayed emotional restraint—or rather, the reduction of women’s full emotional range to a one-note song of uncomplaining cheerfulness—as the only available form of resistance.4 Ehrenreich argues that in the modern world, this emphasis on ‘positive thinking’ has been mobilised to make patients with breast cancer more accepting of their diagnosis and more compliant with punitive treatment regimens; in the 18th century, as we will see, it may also have encouraged female subservience within the household—even when that subservience was seen as contributing to women’s vulnerability to the disease in the first place.

## Cancer and the emotions in the long 18th century

In her seminal work on cancer metaphors in *Illness as Metaphor*, Susan Sontag famously contended that cancer was associated with emotional repression. According to Sontag, ‘many people believe that cancer is a disease of insufficient passion, afflicting those who are sexually repressed, inhibited, unspontaneous, incapable of expressing anger’.5 In 18th-century literature, however, cancer was typically associated not with emotional deficiency but with emotional excess. It was often used as a metaphor for the *dangers* of feeling—of feeling too intensely or for too long, even if the feeling in question might otherwise be considered pleasurable, virtuous or just.

While cancer as discussed in 18th-century texts does not consistently map on to the distinct neoplastic condition we refer to as cancer today, the connection with emotion is integral to the disease’s medical history. From the 16th through to the 18th century, the terms ‘cancer’ and ‘canker’ were used to refer to any kind of persistent, non-healing sore, and medical practitioners connected both ‘cancers’ and ‘cankers’ with emotional states of long duration. As Alanna Skuse and Marjo Kaartinen have noted, many early modern medical writings attributed the development of cancer to the indulgence of grief.6 Skuse observes that some 16th-century and 17th-century medical practitioners also viewed ‘anger, brooding, and mourning’ as potentially ‘contributing to the development of cancers in both sexes’.7

Although medical writers differed somewhat in their views on the nature and causes of cancer, many 18th-century medical texts traced the development of the disease back to the continuation of some sore, cyst or inflammation that had failed to heal.8 In this context, violent, long-lasting emotions could be understood not just as a potential metaphor for the intensity and duration of the cancer-causing injury, but also as a potential cause of the transition from sustained inflammation to life-threatening tumour: as Marjo Kaartinen observes, medical writers like Bryan Cornwell contended that ‘Sorrow, and other disturbance in the mind, easily converts a schirrhus [cyst] into a cancer’.9 Equally, those prone to fits of intense emotion were seen by some practitioners as being at a higher risk of developing cancer, and more difficult to cure if they contracted it: as the surgeon Richard Guy explained of patients with breast cancer, ‘the Dull, Melancholy, Peevish, and Passionate, are more difficult to be relieved, than the Lively, Chearful, Easy and Placid’.10

Within 18th-century literary texts, cancer was often depicted not just as a potential *consequence* of emotions like grief and anger, but also as a metaphor for these feelings and their destructive effects on the self or on society. Particularly in the first half of the century, cancer was a commonplace comparative for strong, lasting feelings with the potential to intensify over time. In this context, the metaphoric link between cancer and emotion ran parallel to a 16th and 17th-century connection—documented at length in Skuse’s *Constructions of Cancer in Early Modern England*—between cancer and predatory or necrophagic animals.11 Just as tumours were described as ‘biting’ or ‘pinching’ their victims like a poisonous snake or crab, ‘devouring’ the flesh like a ravenous wolf, or ‘gnawing’ on the slowly decaying body like a worm, so literary texts described unregulated emotions as ‘consuming’ those who felt them. The same language of ingestion ran through descriptions of both phenomena, with feelings ‘eating’ the heart or ‘feeding’ on their victims. The concept of pain also, of course, linked certain emotions with cancer, as the same terms that were used to describe excruciating cancerous growths could also be applied to feelings that had been allowed to ‘rankle’ or ‘fester’ over time.12

Such language is readily apparent in 18th-century literary descriptions of jealousy, for example. In the 1707 drama *An Evenings Intrigue*, a Mr Love-good advises his less-virtuous peers Mr Pretense and Mr Rattle to cherish their wives, ‘and never to suffer that Monster, *Jealousie*, to approach your Thoughts lest, like a *Cancer*, it eat into your Hearts, and turn your dearest Pleasures into Pain and Sorrow’.13 Similarly, in the 1732 secret history *The Fair Concubine*, envy of a sexual rival is compared with cancer, as the protagonist—the ‘beautiful Vanella’—encourages her peers to retire for the evening rather than see her singled out for attention by the desirable Prince Alexis: ‘*Ladies*, as such a Sight would put you into the most bitter Agonies, I advise you to retire in Time. Envy is a corroding Cancer in the Mind, and furrows many a handsome Face even before the Autumn of Life draws near’.14 In both of these examples, sexual jealousy has the power to consume the victim from within, and in both, the onus of responsibility is placed on the jealous party, with suspicious lovers urged to avoid situations that might provoke such ‘cancerous’ feelings in the first place.

Indeed, prevention is not merely preferable to cure, but appears to be the only possible form of treatment, as such ‘malignant’ feelings are often portrayed as incurable. Thus Eliza Haywood’s *The Perplex’d Dutchess* (1727), for example, describes how the heroine’s envy of a prince named Theanor grows increasingly dangerous without an outlet for expression, and ‘like a Cancer eat[s] into her Heart, and prey[s] upon her very Vitals’.15 Similarly, in Joseph Addison’s 1717 translation of the story of Aglaurus from Ovid’s *Metamorphoses*, unchecked jealousy proves fatal to the eponymous heroine when she attempts to thwart a romantic rendezvous between her sister and the god Mercury.16 While the original myth dramatises Aglaurus’ punishment as a kind of petrification—a literal hardening of the heart—Addison’s translation locates the danger in the heroine’s unregulated passions by introducing the comparative of cancer:

As when a Cancer in the Body feeds,

And gradual Death from Limb to Limb proceeds;

So does the Chilness to each vital Part

Spread by degrees, and creeps into her Heart;

‘Till hard’ning ev’ry where, and speechless grown,

She sits unmov’d, and freezes to a Stone.

But still her envious Hue and sullen Mien

Are in the Sedentary Figure seen.17

Here, as in Haywood’s text, the zoomorphic image of cancer as ‘feeding’ on the body is used to emphasise the dangers of allowing ill feelings to flourish unchecked.

Vanity and pride are similarly portrayed as growing within the heart ‘like a cancer’. In Sir Roger L’Estrange’s 1667 English translation of the satiric ‘visions’ of Francisco de Quevedo, for example, the narrator warns that ambition can be its own form of punishment, as those who are ‘*Cavaliers* and *Signeurs* already…have an Itch upon them to be *Princes:* A vanity that gnaws them like a *Cancer*’.18 Many moralising texts from the period likewise condemn ‘the cancer of Self-love’ or the ‘foul cancer’ of pride.19 In all of these works, as in the accounts of envy and jealousy, the victim bears at least part of the blame for his or her own feelings, with the self-destructive effects of emotion meting out some (implicit or explicit) punishment for the deadly sins of envy and pride.

As Barbara Rosenwein has observed, many early modern ‘emotional communities’ identified particular feelings with states of virtue or of vice, and 18th-century literary comparisons of cancer with all seven of the deadly sins—not just envy and pride, but also avarice, wrath and even sloth—emphasise this need for emotional regulation.20 Eighteenth-century religious texts use the metaphor to warn against the invasion of ‘bad thoughts’ of any kind, with the Welsh vicar and poet Rhys Pritchard, for example, advising readers to ‘Cut out the cancer, e’er it spread,/ Quash bad thoughts, e’er they run a-head’.21

## Cancer and positive feelings

While all of the comparisons between cancer and emotion that I have outlined thus far are broadly in keeping with today’s understanding of cancer—and of states like anger, avarice and jealousy—as undesirable and destructive, Restoration and 18th-century texts *also* used the metaphor of cancer to represent feelings that might in other circumstances be regarded as beneficial or—to use Ehrenreich’s term—‘positive’. These comparisons potentially complicate the distinction that can be identified in many 18th-century theoretical texts, according to Thomas Dixon, between ‘troubling desires and passions on the one hand and milder affections and sentiments on the other’, as 18th-century literary conceptions of cancer suggest that even morally virtuous feelings might have a potentially destructive side.22

Consider, for example, the 17th-century and 18th-century comparisons between cancer and love. In John Bengo’s dramatic poem ‘The Dream of St. Cloud’ (1797), for example, the hero describes a friend’s attempt to ridicule his lovelorn state as ‘like an operation on a cancer’: although the teasing is ‘kindly meant’, it cannot ‘cure’ him of his affliction. ‘Yes’, he laments, ‘I have been allur’d by love’.23 Similarly, in the popular ballad ‘William and Margaret’, the eponymous heroine sickens and dies from the ‘cancer’ of her love for a man who betrays her:

But love had, like the cancer-wor[m],

Consum’d her early prime:

The rose grew pale, and left her cheek;

She dy’d before her time.24

Historical dramas and satires from the period make similar use of the comparative in depicting women blinded by affection: Queen Elizabeth’s love for her sister Mary Queen of Scots is described in John Banks’ 1704 play *The Albion Queens* as the ‘hugg’d cancer’ that must be ‘parted from her breast’, and Marvell’s *Last Instructions to a Painter* (1667) mockingly exposes ‘love’s hid cancer’ within the Duchess of York’s ‘soft breast’.25

While all of these examples use cancer as a metaphor for love that has been bestowed on someone unworthy, unwilling or inappropriate, other texts suggest that even righteous and reciprocated affection can turn carcinogenic if allowed to distract the lover from more important concerns: in John Savage’s 1697 translation of Antonio de Guevara’s *Spanish Letters*, for example, love is described as ‘natural to both Sexes’ and the desire to be loved as ‘agreeable’—but Guevara also likens love to ‘a hidden Cancer’ that ‘fixes not on the Face where it may be seen, nor pulse where it may be felt; but on the poor Heart, where, tho it be very pungent, yet none dares discover it’. Advising no man to ‘trust another, or rely upon himself’ where love is at stake, Guevara reiterates the prevention-better-than-cure wisdom, insisting: ‘After all, the best Remedy I can think of against Love is, that it be not permitted to take root in the Mind’.26 Similarly, a 1703 satire condemns love as an ‘o’re-spreading Cancer’ that ‘knaws the Heart’, rendering the victim oblivious to everything but the ‘pleasure’ it brings.27 These texts warn that even the most seemingly benign of emotions can prove ruinous if allowed to ‘overspread’ acceptable bounds.

## Breast cancer and female feeling

As the 18th century progressed, the commonplace literary descriptions of love, jealousy or guilt as ‘like a cancer’ began to appear less frequently—but the link between cancer and the emotions clearly persisted, as mid and late18th-century texts continued to connect the disease with intense emotional states, particularly in relation to cancer of the breast. This specific emphasis on breast cancer is unsurprising, given the prevailing medical thought at the time: as Skuse explains, cancer was known to afflict both men and women, but it was understood by medical practitioners throughout the early modern period as ‘paradigmatically a disease of the female breasts’.28 This view may have developed in part because breast cancer was more easily detected and identified *as* cancer than tumours in other areas of the body would have been—but it may also have been a product (and, perhaps, a perpetuator) of the stereotypical conception of women as more emotionally labile than men.29 According to Heather Meek, ‘women were thought to be innately disorderly and excessively passionate, qualities that made them susceptible to all illnesses, including breast cancer’.30

Accordingly, just as 18th-century writers used the comparison of feeling with cancer to warn of the dangers of unregulated emotion, so they made breast cancer a vehicle for exploring the particular dangers of feeling for women. Within 18th-century fiction, the relationship between breast cancer and emotion was forged not just by descriptions of cancerous love, grief or jealousy within a woman’s breast, but also by the use of plotlines that portrayed the disease as a direct consequence of uncontrolled emotion. In these texts, breast cancer was not just a metaphor for feeling, but a real-life outgrowth of it—a physiological manifestation of psychological distress.

In Richard Gwinnett’s 1731 semifictionalised memoir *Pylades and Corinna*, for example, a woman’s development of breast cancer is attributed to her overindulgence of intense feelings of loss, as we are told that Corinna’s mother ‘abandoned her self to Grief, which soon occasioned a Cancer in her Breast’.31 Guilt is portrayed as having similar carcinogenic potential: in the 1757 novel *The History of Miss Sally Sable* (1757), the heroine’s would-be adoptive mother nurses long-standing feelings of remorse over having abandoned her young infant charge at the insistence of her ‘imperious Husband’. Years of concealment cause emotional distress that culminates in the development of a fatal breast tumour.32 In these and other texts, the link between cancer and feeling serves a clear cautionary purpose, with the breast cancer plotline used to condemn the angry, grieving or angst-ridden woman.

And just as 18th-century writers connected the development or growth of breast cancer with emotional turmoil, so they portrayed emotional restraint—not just stoicism, but a kind of determined, wilful cheerfulness akin to the ‘Smile or Die’ rhetoric condemned by Ehrenreich—as the best possible response to the illness. Here, once again, the distinction between dangerous ‘passions’ and benevolent ‘affections’ seems to have played an important but ultimately inconsistent role, as fictional narratives depict violent feelings of sorrow or guilt as (literally and metaphorically) carcinogenic, while promoting carefully regulated happiness as the breast cancer sufferer’s sole means of resistance or redemption.33

Thus in the 1779 novel *The Wedding Ring; Or, History of Miss Sidney*, for example, the hero praises the ‘smiling composure’ with which his dying aunt endures the pain from ‘an inveterate putrid cancer in her breast’.34 The cheerfulness of this ‘dying saint’ (as he terms her) proves essential to ‘the uncommon fortitude with which she bears the most painful operation the art of surgery can inflict’—as well as the long, slow decline that follows her unsuccessful mastectomy.35 Similarly, in ‘The Pleasing Story of Master Want-thought’ (1795), the eponymous hero gets an unexpected lesson in stoicism when he and his friend, Master Nevill, meet a young girl whose grandmother has ‘a cancer in her breast, which was extremely painful, and it was thought would soon occasion her death’.36 Despite suffering from a tumour so ‘exceedingly offensive’ that her neighbours shun her, the grandmother maintains an attitude of cheerful compliance, calmly awaiting the moment when she will be able to ‘receive her reward’ for always being ‘so patient, and always [doing] her duty so well’.37 The ‘reward’ here, of course, is death.38

Crucially, in many such tales of suffering female virtue, ‘doing one’s duty’ involves not just emotional self-reliance, but continued contribution to the household economy. Many 18th-century portrayals of breast cancer emphasise financial hardship: despite the lack of connection with hygiene, breast cancer is routinely portrayed in the mid to late century as a condition correlated with, or exacerbated by, poverty. Considered in this context, idealised portraits of cancer-stricken women may have served an economic as well as a social purpose: if, as Ehrenreich suggests, the emphasis on ‘positive thinking’ today has made women more accepting of the disease and its punitive treatment regimens, then 18th-century exhortations to ‘smile or die’ may have encouraged ailing wives and mothers to continue supporting the household while minimising their own demands on the family’s financial and emotional resources. (In this sense, the affective self-control demanded of breast cancer sufferers might even be considered a form of work in its own right—an ‘emotional labour’ akin to the feigned happiness demanded of those who work in today’s service industry.)39

Perhaps the most influential example of this industrious virtue appears in Hester Chapone’s 1752 moral tale *Fidelia*. First published in John Hawkesworth’s mid-century periodical *The Adventurer*, *Fidelia* tells the story of an erring young woman who finds redemption through the moralising conversation of a grieving mother who is dying of breast cancer. The narrative of Fidelia’s early years follows the conventional storyline of what was to become known in the 19th century as the ‘fallen woman’: seduced and abandoned by an unscrupulous lover, Fidelia finds herself bereft of friends, reputation and financial support. Driven to despair, she is about to throw herself into a river when an elderly clergyman who hears her lamentations leads her back to his home to meet his wife, ‘a middle aged woman, pale and emaciated, but of a cheerful, placid countenance’.40 After relating Fidelia’s story to her, the clergyman tasks his wife with teaching their despondent guest something about the power of positive thinking:

‘This poor lady’, said he, ‘from the fault of her education and principles, sees every thing through a gloomy medium: she accuses Providence, and hates her existence for those evils, which are the common lot of mankind in this short state of trial. You, my dear, who are one of the greatest sufferers I have known, are best qualified to cure her of her faulty impatience, and to convince her, by your own example, that this world is not the place in which virtue is to find its reward. She thinks no one so unhappy as herself; but if she knew all that you have gone through, she would surely be sensible, that if you are happier than she, it is only because your principles are better’.41

These remarks identify women’s happiness as entirely a matter of ‘principle’, with the clergyman’s comparison between Fidelia and his wife implicitly assuming that emotions are always within the emoter’s control. By this measure, the chief danger to Fidelia’s well-being is not the very real threat of poverty or the social ostracism she faces, but rather, the ‘gloomy’ attitude that makes her assume ‘no one [is] so unhappy as herself’.

Unsurprisingly, the clergyman’s wife agrees with this assessment, issuing an endorsement of emotional regulation that champions wilful happiness as the source of her own continued strength in a life filled with pain and suffering:

‘Indeed, my dear madam’, said she, ‘that is the only advantage I have over you; but that, indeed, outweighs every thing else. It is now but ten days since I followed to the grave my only son, the survivor of eight children, who were all equally the objects of my fondest love. My heart is not less tender than your own, nor my affections less warm. For a whole year before the death of my last darling, I watched the fatal progress of disease, and saw him suffer the most amazing pains. Nor was poverty, that dreaded evil to which you could not submit, wanting to my trials. Though my husband is by his profession a gentleman, his income is so small, that I and my children have often wanted necessaries: and though I had always a weakly constitution, I have helped to support my family by the labour of my own hands. At this time I am consuming by daily tortures, with a cancer which must shortly be my death. My pains, perhaps, might be mitigated by proper assistance, though nothing could preserve my life; but I have not the means to obtain that assistance’.42

Here once again, breast cancer is connected with intense emotion, as the tumour develops in conjunction with—perhaps even as a consequence of—the mother’s ‘fondest love’ for her children and subsequent grief over their deaths. (The clergyman’s wife takes care to emphasise that her ‘heart is not less tender’ than Fidelia’s, ‘nor [her] affections less warm’; it is simply that she, unlike her self-pitying guest, has learnt how to control such feelings.)

If the emotional strains of motherhood and bereavement have contributed to the development of the wife’s illness, then emotional restraint now seems essential to resisting its spread: Fidelia expresses her astonishment at the ‘appearance of chearfulness and serene complacency which shines so remarkably in [the wife’s] countenance, and animates every look and motion’.43 Once again, ‘chearfulness’ and ‘complacency’ are championed as essential female virtues, enabling the cancer-stricken mother not just to stave off death, but—perhaps more importantly—to continue supporting her family ‘by the labour of [her] own hands’.

Some reworkings and imitations of Chapone’s *Fidelia* make even more of this central message ‘that sufferings and happiness are not incompatible’, with an adaptation for young ladies by Jeanne-Marie Le Prince de Beaumont, for example, playing up both the horror of the disease—in this version, the wife removes her clothing to show Fidelia the ‘cancer that eats [her] up alive, attended with pains beyond expression’—and the power of emotional self-mastery: declaring herself to be ‘by nature weak, impatient, and touchy’, the wife explains that she has managed to quash such passions, maintaining instead ‘a pure and unaltered chearfulness’ that ‘banishes all dread, vexation, and despair’.44

A 1788 verse adaptation goes further still, suggesting that breast cancer should be embraced as an act of divine will. In this retelling, the clergyman’s wife has not only learnt to bear her pain without complaint, but to love the source of that pain as a supreme being who ‘wounds’ her for her own good. The clergyman describes his wife’s condition to Fidelia:

A cancer now her feeble frame devours;

Too ill our scanty means her wants supply,

The nurse’s care, and physic’s aid deny.

Yet mark, her breast no fretful murmur moves;

The Pow’r that wounds her, she adores and loves.45

Here the rhetoric comes close to that of today’s self-help books encouraging cancer sufferers to see their condition as a gift. Such works encourage ‘positive thinking’ by contending that cancer can make patients more mindful of the advantages they ordinarily enjoy—but, as Ehrenreich observes, the discourse of cancer as gift also has the potentially pernicious effect of silencing expressions of anger and other ‘negative’ emotions when such outbursts might be therapeutically or politically useful.46 Certainly, in the 1788 poem, this repressive approach proves effective, as Fidelia begs the dying woman to teach her those principles that will help her ‘to control’ her own feelings of pride and doubt—even though those feelings seem entirely justified.47

## Breast cancer, women’s roles and the sentimental novel

While texts like Chapone’s potentially endorse the oppression of women by encouraging female submission to pain and suffering, they also draw attention to women’s vulnerability to illness and abuse in the first place, by connecting the development of breast cancer with women’s roles within the household. Even *Fidelia*, with its lengthy glorification of female emotional self-mastery, nonetheless acknowledges that the wife’s condition has been exacerbated (if not precipitated) by her duties as a wife and mother.

Other 18th-century literary texts make much more of this connection, complicating the basic plotline of the woman who perishes of cancer because she ‘succumbs’ to her feelings by identifying and expanding on the larger social forces—often male-driven forces—that lead women to experience emotional distress in the first place.48 In some fictional works a female character’s development of breast cancer is directly attributed to an act of male cruelty: the 1769 tale ‘The Lottery Ticket’, for example, describes how an ‘old debauched lord’ gives his innocent young bride ‘a blow upon her breast, in a fit of jealousy, which brought on a cancer; and that cancer in less than a twelvemonth, during which her sufferings in body and mind are not to be described, brought her to the grave’.49 Similarly, in the 1754 play *The Female Parliament*, a young woman contracts breast cancer after receiving ‘a Kick in the Breast’ from a misogynistic nobleman.50 Storylines of this sort present the disease as a direct consequence of male violence, locating women’s emotional fragility within the politically complicating context of spousal or other male abuse.51

On a similar principle, the links between breast cancer and motherhood—dramatised in many later 18th-century novels and poems, including *Fidelia*—once again undermine the divide between dangerous ‘passions’ and virtuous ‘affections’, reminding us that even that most benevolent of the domestic feelings, maternal love, can prove carcinogenic.52 Like the accounts of cancer arising from spousal abuse, these works connect the disease with women’s work, establishing an association between the demands placed on women by marriage or motherhood and their particular susceptibility to cancer.

In Maria Smyth’s 1783 novel *The Woman of Letters; or the History of Miss Fanny Belton*, for example, the heroine is introduced to a young mother whose fatal breast tumour develops because of, rather than in spite of, her virtuous behaviour within the household. In this sentimental narrative, the eponymous heroine—a clergyman’s daughter left in impoverished circumstances and sent by her conniving aunt to find work in London—is saved from self-pity by a timely comparison between her ‘own most helpless situation’ (as she initially sees it) and the far more difficult circumstances of a local woman named Mrs Perry, who faces a painful, lingering death from breast cancer.53

As this summary suggests, many details in the episode are reminiscent of Chapone’s moral tale. Like the clergyman’s wife, Mrs Perry is an idealised figure, her suffering providing the novel’s heroine with ‘a lesson of industry [and] of patience’.54 And like the wife in *Fidelia*, Mrs Perry continues to labour despite her illness, ‘sit[ting] with such patient resignation at her work,—earning bread for her little ones, day after day, and under the pressure of such a painful disorder, such a shocking, incurable malady’.55 Just as the clergyman’s wife is introduced as ‘a middle aged woman, pale and emaciated’, so Mrs Perry is introduced as ‘an amiable young woman—pale—and—emaciated.—“Want, *staring* in her haggard eye.”'56 And when Fanny and her landlady, Mrs Williams, visit the dying mother and enquire after her health, Mrs Perry’s reply reflects that same combination of superhuman stoicism and wilful cheerfulness endorsed throughout Chapone’s narrative:

‘I thank heaven’, answer’d the patient sufferer, ‘I have had a tolerable night for *me*, madam’—looking up with a sweet smile.57

Once again, the cancer-stricken mother is a model of emotional restraint, her ‘patient resignation’ bolstered by ‘hope’ and ‘faith’ (terms that remain watchwords in today’s ‘pink ribbon’ culture).58 In fact, Mrs Perry seems to be preparing herself for that very fate most dreaded by Barbara Ehrenreich: to die ‘with a sweet [little] smile’ on her face. She even expresses gratitude for her illness, grasping her Bible and exclaiming: ‘It is *good* for me that I have been in *trouble*, that I may learn [God’s] statutes’.59

Yet while Fanny is initially sobered by the comparison, feeling ashamed that she herself should ‘dare to complain’ when Mrs Perry does not, Smyth’s novel ultimately presents a far less optimistic account of female agency—and thus a far more ambiguous account of the politics of breast cancer—than *Fidelia*. For one thing, we are directly informed that Mrs Perry’s situation is the result of her husband’s cruelty. Fanny’s landlady explains:

Her husband, I understand, is a most vile wretch, and has left her absolutely to starve with these poor children—the youngest not a year old.—The brutality of her husband has been the cause of this dreadful malady in her breast; a cruel blow occasioned it.60

Here the delinquent Mr Perry is implicated as both the cause of his wife’s illness and an intensifier of its evils, his abandonment dooming her to an impoverished life in which she cannot afford to stop working or to purchase pain relief.

Mrs Perry’s sufferings are also exacerbated by her duties as a mother: although there is no insinuation here that her illness might have been caused by childbearing, there seems little doubt that her children make her situation more difficult, not only by obliging her to continue working, but also by compelling her to put on a ‘brave face’ to spare them from pain and worry. And given that Fanny herself later falls victim to similar circumstances—she marries a man who runs off to France with another woman, leaving her and her daughter to languish in prison for his debts—it becomes increasingly difficult to see the novel’s portrait of Mrs Perry as an exhortation to female emotional self-mastery rather than as an indictment of male misbehaviour.

Indeed, *Fanny Belton*’s tragic conclusion highlights another crucial element in late-18th-century fiction that potentially complexifies the exemplarity of the breast cancer narrative: the fashion for literature designed to invoke intense feeling—often, though not exclusively, in a female readership—by depicting scenes of virtue in distress.61 In Smyth’s story, as in other sentimental novels of the period, the mother with breast cancer is not just a model of goodness, but an object for reader sympathy: Mrs Perry’s circumstances are made more noble by the suppression of her emotions, but they are paradoxically designed to *induce* emotion in others. These intentions potentially destabilise the conservative social politics of the links between emotion and cancer, for while sentimental novels reiterate the need for wilful cheer in the cancer-stricken woman, they identify grief as an appropriate, even desirable, response on the part of the sympathetic female observer.62

This shift explains why Smyth’s text idealises Mrs Perry for her emotional restraint but champions the heroine for having a ‘heart ever open to a tale of woe’—and indeed, both Fanny and Mrs Williams shed copious tears over Mrs Perry’s unhappy situation.63 The dying mother herself is allowed to succumb to tears on only one occasion, her momentary lapse into self-pity inviting the reader to indulge in those same expressions of grief she denies to herself. Mrs Williams recalls:

I call’d into her room yesterday morning, and for the first time saw her weeping.—‘I am ashamed of these tears, Madam’ (said she forcing a sweet languid smile) ‘but my little Billy, here, has been telling me I shall not die’.—‘No, my poor mamma shall not die—shall she, Mrs. Williams’, (said the pretty fellow) ‘for what shall such a little baby, as sister Sally do, and I do, without her?’

The wretched mother cast her eyes to heaven! and with a silent, earnest address, seem’d to implore its aid for her precious children.64

Here Mrs Perry’s tears establish her as a loving mother—but her attempts to suppress them ultimately work to encourage, rather than caution against, an emotional response from the reader, as she practises that ‘recollection and self-command’ identified in Adam Smith’s *Theory of Moral Sentiments* as crucial to obtaining the sympathy of others.65

Perhaps most importantly of all, Smyth’s novel embeds the narrative of Mrs Perry’s suffering within the larger story of Fanny’s own unhappy marriage and untimely death. (As the novel’s ‘editor’ observes, it is just as well that the story of Mrs Perry should remain brief, for ‘Our poor heroine *herself* will, in due time, claim the tribute perhaps of a tear from the reader’.66) Confined within the Fleet Prison for her husband’s debts, Fanny is initially able to remain ‘calm’ and ‘compos’d’, taking ‘great comfort from [her] devotions;-- [her] bible, and [her] child’—yet this emulation of Mrs Perry’s example is soon defeated by the cruelty of Fanny’s circumstances. After her daughter sickens and dies, Fanny loses first her sanity and then her will to live, finally expiring in a filthy jail cell, ‘emaciated’ and delirious from consumption.67

To emphasise that Fanny’s fate—much like Mrs Perry’s—results from unbridled male cruelty rather than unregulated female emotion, the ‘editor’ of Fanny’s letters warns female readers against ‘too *precipitately* enter[ing] the married state’, and her concluding reflections on ‘the fate of [an] unfortunate virtuous woman’ locate the blame for Fanny’s death squarely on the shoulders of her husband—aided, perhaps, by the patriarchal institutions that enable men to mistreat women with impunity.68 In a brief peroration, the editor invites female readers to combat such mistreatment—*not* by practising emotional restraint, but rather, by sympathising with the plights of other women:

Let your susceptible hearts be touch’d with compassion, at least for your *own* sex, who, many of them, from unavoidable *misfortunes*, are at once *innocent* yet *wretched*.69

These concluding lines urge readers to share and ameliorate the burden of female suffering, rather than stoically accepting or embracing it.

Similar motives also seem to underpin Anna Maria Bennett’s sentimental portrayal of a dying mother in her 1789 novel *Agnes de-Courci*. In this melodramatic tragedy, the hero’s adoptive mother, Ann Montford, faces a slow, lingering death from breast cancer despite a lifetime of exemplary behaviour, and her final ‘confession’—outlined in a letter to her friend Mary Moncrass—establishes her as a victim of male cruelty.70 Like Fanny Belton and Mrs Perry, Mrs Montford has bestowed her love on an unworthy object: in youth, she confesses, she had entertained an ill-judged, but entirely chaste, attraction to an unscrupulous young man named Mr Neville. Although she fled Neville’s company after learning of his seduction and abandonment of another young woman, Montford reveals that she has continued to love him from a distance, and has secretly raised his illegitimate son as her own, ‘cherishing [her] first passion for Neville, while [she] indulged [her] fondness for [his son] Edward’.71 Her letter is thus a revelation of love rather than an exposure of wrongdoing—‘an old maid’s confession’ for which, as she herself observes, her friend might accuse her of folly, but must ‘acquit her of guilt’.72

Montford’s revelations confirm that breast cancer can be a punishment visited on the undeserving, even those who quash inappropriate feelings. Like Mrs Perry, she recognises that her tumour will kill her—she remarks that ‘the formidable advances it now makes on my constitution, and the impossibility of repelling it, are omens of a speedy dissolution’—but she decorously resolves to accept her fate rather than pursuing the mastectomy that might save her life: ‘my surgeon talks of the knife, but I will not submit to the operation, and my complaint is mortal’, she declares.73 Like Mrs Perry, she bears her suffering with superhuman stoicism, not only remaining silent on the subject of her own physical pain, but apologising for the ‘trouble’ that her letter must cause her correspondent.74

Here once again the breast cancer sufferer displays a saintlike composure, accepting her death without objection or anger—but here, as in *Fanny Belton*, the story of female suffering is ultimately used to invoke, rather than repress, expressions of feeling. Because Montford’s confession letter reveals Edward’s true parentage, it provides the catalyst for the novel’s central tragedy, leading Edward to discover that the woman he has just married is his half sister. Horrified by this revelation, Edward tears out his own hair and then takes his own life, leaving his tormented wife, the eponymous Agnes de-Courci, to follow him to the grave. Seen in this larger context, the story of Ann Montford’s cancer becomes something like an emotional hors d’oeuvre, effectually whetting the reader’s appetite for the still more melodramatic feast to follow. As with the account of Mrs Perry in *Fanny Belton*, we are invited not to *learn* from the breast cancer sufferer’s miseries, but to *feel* for them. It is a distinction with important potential consequences for the novel’s politics—but even here, such expressions of emotion are only allowed to go so far, with grief identified as an acceptable response in the outside observer, but not in the cancer-stricken woman herself.

## Coda: the 19th century and beyond

While sentimental novels like *Fanny Belton* and *Agnes de-Courci* opened up a space for the potential expression of women’s ‘negative’ feelings around breast cancer, that space was soon after closed off by what the historian William Reddy has described as a change in ‘emotional regime’: as Reddy’s work has demonstrated, the 19th century saw the radical sentimentalism of the 1780s and 1790s replaced by a far more circumspect and conservative political and literary culture.75 This shift helps to account for the relative silence about breast cancer in 19th-century literature: where mid to late18th-century novels describe the condition openly and (sometimes horrifyingly) explicitly, Romantic and Victorian novels begin what Ehrenreich identifies as the euphemising of the disease as ‘a long illness’, with very few female characters dying of breast cancer per se, but several—like Mrs Hale in Gaskell’s *North and South*, for example—consigned to a slow decline from some mysterious, unspecified feminine complaint.

While the confessional mode of the 20th and the 21st centuries has returned us to a more open discussion of breast cancer, it seems also to have returned us to the more repressive gender and social politics that originally accompanied that discussion. In the self-help texts of today, as in the literary texts of the 1700s, responsibility for well-being is placed on the patient herself, with the demand for ‘positive thinking’ narrowing the space for expressions of anger at the injustice of rising breast cancer rates. And ultimately, even in the 18th century, expressions of sentimental distress over the breast cancer sufferer’s fate end up being channelled into charitable action: *Fanny Belton*’s ‘editor’ concludes the text by exhorting those ‘who are bless’d with riches’ to ‘make the compassionate trial’ of visiting and relieving those who are ‘pining in sickness,—and a prey to famine’.76

The same emotional asymmetry characterises breast cancer discourse today, as those with the disease are told to remain optimistic and cheerful, while those around them are encouraged to channel their feelings into supporting the business—and, as sociologists like Samantha King have demonstrated, it is a very big, and often a male-dominated, business—of breast cancer fundraising.77 Now, as in the 18th century, there is an economics as well as a politics to this emotional regime—and although Sontag’s theory that cancer is a disease of ‘insufficient emotion’ suggests that we have lost the centuries-old belief in unregulated emotion as a cause of or contributor to breast cancer, we should continue to consider the extent to which emotional self-control still plays a powerful role, not only in the discourse of cancer, but in the effects of that discourse on the lives and experiences of patients.78

## Notes

1. Barbara Ehrenreich (2009), *Smile or Die: How Positive Thinking Fooled America and The World* (London: Granta), 23, 27–8, 33.

2. Ehrenreich, 17.

3. Ehrenreich, 21.

4. As this essay is concerned with the literary representation of feeling rather than the historical experience of it, I use the 18th-century terms ‘passion’, ‘affection’, ‘emotion’ and ‘feeling’, as opposed to ‘affect’—now often reserved for the non-discursive bodily aspects of feeling (a racing pulse, tears, blushing and so on). For a useful overview of this distinction, see Stephanie Trigg (2014), “Emotional Histories—Beyond the Personalization of the Past and the Abstraction of Affect Theory,” *Exemplaria* 26, no. 1 (Spring): 5–7.As Thomas Dixon notes, the term ‘emotion’ changed in meaning over the course of the 18th century, initially referring to ‘the bodily stirrings accompanying mental feeling’ but subsequently moving ‘from the bodily to the mental domain’ from the mid-century onwards; accordingly, in this essay, I use the term in its mid to late18th-century sense, either as referring to ‘mental feeling’ or as ‘a stylistic variant for central theoretical terms such as ‘passion’ and ‘affection'’. See Thomas Dixon (2012), “‘Emotion’: The History of a Keyword in Crisis,” *Emotion Review* 4, no. 4: 340; see also Thomas Dixon (2003), *From Passions to Emotions: The Creation of a Secular Psychological Category* (Cambridge: Cambridge University Press).

5. Susan Sontag (1990), *Illness as Metaphor* and *AIDS and Its Metaphors* (New York: Anchor), 21. As Clark Lawlor notes, Sontag contrasts the condition with consumption, yet consumption, too, had a complex and changing history over the course of the 18th century, and was at times speculatively connected with cancer in 18th-century medical texts. See Clark Lawlor (2006), *Consumption and Literature: The Making of the Romantic Disease* (Basingstoke: Palgrave), 3, 172.

6. Alanna Skuse (2015), *Constructions of Cancer in Early Modern England: Ravenous Natures* (Houndmills: Palgrave), 58; Marjo Kaartinen (2013), *Breast Cancer in the Eighteenth Century* (London: Pickering and Chatto), 18.

7. Skuse, 58.

8. See, for example, Herman Boerhaave (1715), *Boerhaave’s Aphorisms: Concerning The Knowledge and Cure of Diseases*, trans. and ed. J. Delacoste (London), 113; John Arbuthnot (1732), *An Essay Concerning the Nature of Aliments* (London), 355; Samuel Sharp (1739), *A Treatise on the Operations of Surgery* (London), 48; John Allen (1749), *Synopsis Medicinæ,* 2 vols (London), 2:157; William Norford (1753), *An Essay on the General Method of Treating Cancerous Tumors* (London), 14; Richard Guy (1762), *Practical Observations on Cancers and Disorders of the Breast* (London), xi, xv*; J. William Rowley (1772), *A Practical Treatise on the Breasts of Women* (London), 22; John Burrows, 1785, *A New Practical Essay on Cancers* (London), 81; Henry Fearon (1790), *A Treatise on Cancers*, 3rd edn (London), 54. While medical texts differed in their accounts of the relationship between schirri (which Marjo Kaartinen identifies as ‘cysts’) and cancer, the two conditions were almost always connected, with schirri routinely identified as potential causes of or precursors to cancer. See Kaartinen, 2.

9. See Bryan Cornwell (1784), *The Domestic Physician; or, Guardian of Health* (London), 525, quoted in Kaartinen, 18. See also, for example, Vonn Störck and Anton Freiher (1762), *An Essay on the Medicinal Nature of Hemlock* (Edinburgh), 277; Robert Whytt (1765), *Observations on the Nature, Causes, and Cure of Those Disorders Which Have Been Commonly Called Nervous Hypochondriac* (Edinburgh), 203; Nicholas Waters (1791), *A System of Surgery. Extracted from the Works of Benjamin Bell of Edinburgh by Nicholas B. Waters, MD* (Philadelphia), 43.

10. Guy, 9, quoted in Kaartinen, 13.

11. On zoomorphic imagery and cancer, see Skuse, 61–73. On cancer as compared with a crab, see Kaartinen, 4.

12. Many medical and historical accounts of breast cancer in the 18th century highlight the pain of the disease and its treatments (most notably mastectomy). On this issue, see Kaartinen, 89–124; Heather Meek (2017), “Frances Burney’s Mastectomy Narrative and Discourses of Breast Cancer in the Long Eighteenth Century,” *Literature and Medicine* 35, esp. 32–4.

13. Anon (1707), *An Evening's Intrigue, a text contained within The Spanish Libertines*, trans. John Stevens (London), 188. The description of jealousy as a ‘canker’ seems to have been a commonplace in Restoration drama. See, for example, Nicholas Rowe (1708), *The Royal Convert* (London), 12; Richard Brome (1731), *The Jovial Crew, A Comic-Opera* (London), 51; Elizabeth Cooper (1735), *The Rival Widows; Or, the Fair Libertine* (London), 107.

14. Anon (1732), *The Fair Concubine: or, the Secret History of the Beautiful Vanella* (London), 35.

15. Eliza Haywood (1727), *The Perplex’d Dutchess: or, Treachery Rewarded. Being Some Memoirs of the Court of Malfy* (Dublin), 37.

16. Jacob Tonson’s 1717 edition of the *Metamorphoses* features translations by John Dryden, Joseph Addison, Samuel Garth and a number of other well-known early-18th-century poets. Addison is known to have translated at least books 2 and 3.

17. Ovid (1717) *Ovid's Metamorphoses in Fifteen Books. Translated by the Most Eminent Hands* (London), 70.

18. Francisco De Quevedo (1667), *The Visions of Dom Francisco de Quevedo Villegas, Knight of the Order of St. James*, trans. Roger L’Estrange (London), 60. See also, for example, William Cook (1783), *The Capricious Lady: A Comedy, (altered from Beaumont and Fletcher) as It Is Now Performing at the New Theatre-Royal* (London), 10.

19. See, for example, Walter Montagu (1648), *Miscellanea Spiritualia: or, Devout Essaies* (London), 288; Theophilus Gale (1672), *The Anatomie of Infidelitie, or, An explanation of the Nature, Causes, Aggravations and Punishment of Unbelief* (London), 28; Anon (1782). *The Beauties of Administration, a Poem. With an Heroic Race to the Palace, between L-d Sh-lb-ne and the Hon. C. J. F-x*. (London), 61.

20. Barbara H. Rosenwein (2016), *Generations of Feeling: A History of Emotions, 600–1700* (Cambridge: Cambridge University Press), esp. 69–71, 74–87, 307–08, 315–16. On avarice as cancer, see, for example, Peter Pindar (1789)(pseud. John Wolcot), *Subjects for Painters* (London), 78; on sloth as cancer, see the oft-reprinted lines from the *Mirror for Magistrates*, quoted, for example, in Thomas Hayward (1738), *The British Muse, or, a Collection of Thoughts Moral, Natural, and Sublime, of Our English Poets: Who Flourished in the Sixteenth and Seventeenth Centuries*, 3 vols (London), 2:72; for rage compared with cancer, see Aristænetus (1716), *Letters of Love and Gallantry. Written in Greek by Aristænetus* (London), 47.

21. Rhys Pritchard (1771), *The Welshman's Candle: or the Divine Poems of Mr. Rees Prichard, Sometime Vicar of Landovery, in Carmarthenshire* (Carmarthen), 191. See also, for example, Johann Casparr Lavater (1788), *Aphorisms on Man: Translated from the Original Manuscript of the Rev. John Caspar Lavater* (London), 99; Joseph Dennie (1796), *The Lay Preacher; or Short Sermons, for Idle Readers* (Walpole [New Hampshire]), 42.

22. Dixon, 339.

23. John Bengo (1797), *Poetry Miscellaneous and Dramatic* (Edinburgh), 85.

24. Anon (1790), *The New Olio: Or, a Collection of Choice Whims* (London), unpag.

25. John Banks (1704), *The Albion Queens: or the Death of Mary Queen of Scotland, as it is Acted at the Theatre-Royal* (London), 58; Andrew Marvell (1963–1975), “Last Instructions to a Painter” in *Poems on Affairs of State*, 7 vols, ed. George de F. Lord (New Haven: Yale), 1:103 (line 74).

26. Antonio De Guevara (1697), *Spanish Letters: Historical, Satyrical, and Moral; of the Famous Don Antonio de Guevara Bishop of Mondonedo,* trans. John Savage (London), 142.

27. Anon (1703), *A Satyr Against Love. Revised and Corrected by Mr. Congreve* (London), 4.

28. Skuse, 26.

29. See Kaartinen, 9–10.

30. Meek, 41.

31. Richard Gwinnett (1731), *Pylades and Corinna: or, Memoirs of the Lives, Amours, and Writings of Richard Gwinnett Esq*., 2 vols (London), 1:lxix.

32. Anon (1757), *The History of Miss Sally Sable*, 2 vols (London), 2:54–5.

33. See Dixon, 339.

34. Anon (1779), *The Wedding Ring: Or, History of Miss Sidney*, 3 vols (London), 2:228–9.

35. Anon, 2:228.

36. Anon (1795), *The British Champion; Or, Honour Rewarded* (York), 78–9.

37. Anon, 81.

38. Breast cancer is almost always portrayed as fatal in 18th-century fiction. For exceptions to this, see, for example, Jean Digard de Kerguette (1757), *True Merit, True Happiness; Exemplified in the Entertaining and Instructive Memoirs of Mr. S-*., 2 vols (London): 1:169-70; Anon (1773), *The Supposed Daughter; or, Innocent Impostor*, 3 vols (London), 2:188. In some works—most notably Maria Edgeworth’s 1801 novel *Belinda*—a woman who is believed to have breast cancer survives because her condition turns out to be something more benign. See Maria Edgeworth (1801), *Belinda*, 3 vols (London), 2:364–5.

39. On women’s ‘emotional labor', see Arlie Russell Hochschild (1983), *The Managed Heart: Commercialization of Human Feeling* (Berkeley: University of California Press).

40. Hester Chapone (1752–1754), *Fidelia,* in *The Adventurer*, 2 vols (London), 2:52.

41. Chapone, 2:52.

42. Chapone, 2:52–3.

43. Chapone, 2:53.

44. Jeanne-Marie Le Prince de Beaumont (1760), *The Young Ladies Magazine, or, Dialogues Between a Discreet Governess and Several Young Ladies of the First Rank Under Her Education*, 4 vols (London), 3:109–10. Interestingly, this version of the narrative associates the development of breast cancer with ‘hard labour and toil’ rather than emotional excess; it also enhances the sufferings of the recently deceased child. These alterations potentially complicate Chapone’s original endorsements of hard work and emotional self-control. I discuss the links between breast cancer and women’s household work in greater detail in the next section of this essay.

45. Anon (1788), *Prolusiones Poetica; or a selection of poetical exercises, in Greek, Latin, and English* (Chester), 178.

46. On the ‘positive embrace’ of breast cancer as a gift, see Ehrenreich, 27–33. On the use of ‘negative feelings’ like anger and depression as ‘a possible resource for political action’, see, for example, Ann Cvetkovich (2012), *Depression: A Public Feeling* (Durham: Duke University Press), 2.

47. Anon,179.

48. In the previously mentioned *History of Miss Sally Sable*, for example, it is the brutality of the breast cancer sufferer’s ‘imperious husband’ that causes her to develop carcinogenic levels of guilt in the first place.

49. Anon, 1769–1796, *The Town and Country Magazine; Or Universal Repository of Knowledge, Instruction, and Entertainment*, 27 vols (London), 1:635.

50. Anon (1754) *The Female Parliament. A Seri-Tragi-Comi-Farcical Entertainment, Never Acted in Eutopia Before* (London), 44.

51. According to Alanna Skuse, women’s susceptibility to breast cancer was identified with ‘social or domestic phenomena which were either sex-specific, or affected women to a greater extent than men’, including ‘maternal nursing, sex, domestic violence and emotional trauma’ (Skuse, 54).

52. As Skuse and Kaartinen have noted, medical texts from the period identified mothers as being at greater risk for breast cancer, and the illness was often directly connected with breast feeding and other aspects of maternal life. See Skuse, 53–9; Kaartinen, 14–6. Kaartinen also notes that breast cancer was linked to childlessness and to menopause; this suggests some potential for divergence between fictional depictions of breast cancer—many of which were penned by women and connected cancer with women’s roles as wives and mothers—and medical conceptions of breast cancer—all of which were written by men, and which connected the disease with childlessness as well as childbearing. See Kaartinen, 12–4.

53. Maria Smyth (1783), *The Woman of Letters; or the History of Miss Fanny Belton,* 2 vols (London), 2:2, 2:15.

54. Smyth, 2:15.

55. Smyth, 2:11.

56. Chapone, 2:52; Smyth, 2:17.

57. Smyth, 2:17.

58. Smyth, 2:12.

59. Smyth, 2:18–9.

60. Smyth, 2:13.

61. On the fashion for sentimental literature and its aims of invoking feeling in a (predominantly female) readership, see, for example, R. F. Brissenden (1974), *Virtue in Distress: Studies in the Novel of Sentiment from Richardson to Sade* (London: Macmillan); Janet Todd (1986), *Sensibility: An Introduction* (London: Methuen); G. J. Barker-Benfield (1992), *The Culture of Sensibility: Sex and Society in Eighteenth-Century Britain* (Chicago: University of Chicago Press). For a good basic overview, see Janet Todd (1988), “The Sentimental Novel” in *The Encyclopedia of the Novel*, 2 vols, ed. Paul Schellinger (Chicago: Fitzroy Dearborn), Literature Online.

62. As Heather Meek has demonstrated, medical texts from this period also betray glimmerings of sympathy, as even enthusiastic promoters of the mastectomy like Henry Fearon condoled with patients over their diagnosis and empathised with their fears about surgery. See Meek, 39–40.

63. Smyth, 2:11, 2:17.

64. Smyth, 2:12.

65. On the importance of self-command, see Adam Smith (1984), part 6, section 3 of the *Theory of Moral Sentiments*, ed. D.D. Raphael and A.L. Macfie (Indianapolis: Liberty Fund), 27. Smith’s text also, of course, praises ‘industry and frugality’ in obtaining the ‘approbation of the impartial spectator’, 314.

66. Smyth, 2:19 n.*.

67. Smyth, 2:273, 2:275.

68. Smyth, 2:278. We are also told that Mrs Perry ‘has been genteely bred, and well-educated; but unfortunately has highly disoblig’d her friends by marriage’, 2:13.

69. Smyth, 2:278.

70. Anna Maria Bennett (1789), *Agnes de-Courci: A Domestic Tale*, 4 vols (Bath): 4:131–42.

71. Bennett, 4:137.

72. Bennett, 4:132.

73. Bennett, 4:131, 4:141. It is worth noting that while many surgeons promoted mastectomy as a treatment for breast cancer in this period, some—like the renowned cancer specialist Henry Fearon—expressed compassion for patients unwilling to endure the procedure. See Meek, 39–40.

74. Bennett, 4:141.

75. William M. Reddy (2001) *, The Navigation of Feeling: A Framework for the History of Emotions* (Cambridge: Cambridge University Press).

76. Smyth, 2:277–8.

77. Samantha King (2006), *Pink Ribbons Inc.: Breast Cancer and the Politics of Philanthropy* (Minneapolis: University of Minnesota Press).

78. While this essay has focused on fictional material, it is worth noting that the language of moralising cheerfulness also inflected portraits of historical figures with the disease.Alexander Pope's epitaph ‘On Mrs. Corbet, Who died of a Cancer in her Breast’, for example, describes breast cancer as a test of female virtue, and concludes that ‘The Saint sustain’d it, but the Woman dy’d’. Similarly, Hester Lynch Piozzi's *Anecdotes of the Late Samuel Johnson* (London) includes a poem praising Piozzi’s mother for her cheerful forbearance during the final months before her death from breast cancer: ‘Th’envenom’d cancer revell’d in her veins; Prey’d on her spirits—stole each power away; Gradual she sunk, yet smiling in decay; She smil’d in hope, by sore afflictions try’d, And in that hope the pious Christian died’. These and other texts—including Fanny Burney’s record of her mastectomy—suggest that fictional accounts of breast cancer may well have had a coercive influence on women suffering from the disease (see Pope, 6:91; Piozzi, 132). On fictive or literary tropes in Burney’s account of her mastectomy, see, for example, Julia L. Epstein (1986), “Writing the Unspeakable: Fanny Burney’s Mastectomy and the Fictive Body,” *Representations* 16, 131–66; Meek, esp. 30–4; Heike Hartung (2007), “’Doleful Ditties’ and Stories of Survival—Narrative Approaches to Breast Cancer in Frances Burney, Maria Edgeworth and Susan Sontag,” *Gender Forum* 19, 49–51; Kay Torney (1991), “Fanny Burney’s Mastectomy,” *Meridian: The La Trobe University English Review* 10, 79–85; John Wiltshire (1993), “Fanny Burney’s Face, Madam D’Arblay’s Veil,” 245–65 in *Literature and Medicine during the Eighteenth Century*, ed. Marie Mulvey Roberts and Roy Porter (London: Routledge), 255–8.

## References

[R1] Allen John Synopsis Medicinæ, vol. 2 vols London: Printed for W. Innys, W. Meadows, R. Manby and H. S. Cox, 1749.

[R60] Anon A Satyr Against Love. Revised and Corrected by Mr. Congreve. London, 1703.

[R29] Anon An Evening's Intrigue, a text contained within The Spanish Libertines, edited by Stevens John, 163–252. London: Printed for Samuel Bunchley, 1707.

[R30] Anon The Fair Concubine: or, the Secret History of the Beautiful Vanella. London: Printed for W. James, 1732.

[R32] Anon The Female Parliament. A Seri-Tragi-Comi-Farcical Entertainment, Never Acted in Eutopia Before. London: Printed next door to the Saddle on the Right Horse, 1754.

[R38] Anon The History of Miss Sally Sable, 2 Vols London: Printed for F. Noble, 1757.

[R70] Anon The Town and Country Magazine; Or Universal Repository of Knowledge, Instruction, and Entertainment, 27 Vols London: Printed for A. Hamilton, 1769–1796.

[R66] Anon The Supposed Daughter; or, Innocent Impostor, 3 Vols London: Printed for F. and J. Noble, 1773.

[R74] Anon The Wedding Ring: Or, History of Miss Sidney, 3 Vols London: Printed for F. and J. Noble, 1779.

[R6] Anon The Beauties of Administration, a Poem. With an Heroic Race to the Palace, between L-d Sh-lb-ne and the Hon. London: Printed for S. Hooper, 1782.

[R56] Anon Prolusiones Poetica; Or a Selection of Poetical Exercises, in Greek, Latin, and English. Chester: Printed for J. Fletcher, 1788.

[R49] Anon The New Olio: Or, a Collection of Choice Whims. London: Printed for Champants and Whitrow, 1790.

[R11] Anon The British Champion; Or, Honour Rewarded. York: Printed for Wilson, Spence, and Mawman, 1795.

[R2] Arbuthnot John An Essay Concerning the Nature of Aliments. London: Printed for J. Tonson, 1732.

[R3] Aristænetus Letters of Love and Gallantry. Written in Greek by Aristænetus. London: Printed for Bernard Lintot, 1716.

[R4] Banks John The Albion Queens: Or the Death of Mary Queen of Scotland, as it is Acted at the Theatre-Royal. London: Printed for Richard Wellington, 1704.

[R5] Barker-Benfield G. J The Culture of Sensibility: Sex and Society in Eighteenth-Century Britain. Chicago: University of Chicago Press, 1992.

[R7] Bengo John Poetry Miscellaneous and Dramatic. By an artist. Edinburgh: Printed by Geo. Reid and Co., 1797.

[R8] Bennett Anna Maria Agnes de-Courci: A Domestic Tale, 4 Vols Bath: Printed by S. Hazard, 1789.

[R9] Boerhaave Herman Boerhaave’s Aphorisms: Concerning The Knowledge and Cure of Diseases. Translated and edited by J. Delacoste. London: Printed for B. Cowse, and W. Innys, 1715.

[R10] Brissenden R. F Virtue in Distress: Studies in the Novel of Sentiment from Richardson to Sade. London: Macmillan, 1974.

[R13] Brome Richard The Jovial Crew, A Comic-Opera. London: Printed for J. Watts, 1731.

[R14] Burrows John A New Practical Essay on Cancers. London: Printed for the Author, 1785.

[R15] Chapone Hester “Fidelia” In The Adventurer, vol. 2, 37–54. London: Printed for J. Payne, 1752–1754.

[R16] Cook William The Capricious Lady: A Comedy, (Altered from Beaumont and Fletcher) as It Is Now Performing at the New Theatre-Royal. London: Printed for C. Dilly, 1783.

[R17] Cooper Elizabeth The Rival Widows; Or, the Fair Libertine. London: Printed for T. Woodward, 1735.

[R18] Cornwell Bryan The Domestic Physician; or, Guardian of Health. London: Printed for the Author, 1784.

[R19] Cvetkovich Ann Depression: A Public Feeling. Durham: Duke University Press, 2012.

[R21] De Guevara Antonio Spanish Letters: Historical, Satyrical, and Moral; of the famous Don Antonio de Guevara Bishop of Mondonedo. Translated by John Savage. London: Printed for A. Roper, 1697.

[R23] De Quevedo Francisco The Visions of Dom Francisco de Quevedo Villegas, Knight of the Order of St. James. Translated by Roger L’Estrange. London: Printed for H. Herringman, 1667.

[R20] Dennie Joseph The Lay Preacher; Or Short Sermons, for Idle Readers. Walpole (New Hampshire: Printed by David Carlisle, 1796.

[R22] Digard de Kerguette Jean True Merit, True Happiness; Exemplified in the Entertaining and Instructive Memoirs of Mr. S-, vol. 2 Vols London: Printed for Francis Noble, 1757.

[R25] Dixon Thomas From Passions to Emotions: The Creation of a Secular Psychological Category. Cambridge: Cambridge University Press, 2003.

[R24] Dixon Thomas “"Emotion": The History of a Keyword in Crisis.” Emotion Review 4, no. 4 (2012), 338–44. 10.1177/1754073912445814 23459790 PMC3573683

[R26] Edgeworth Maria Belinda, 3 Vols London: Printed for J. Johnson, 1801.

[R27] Ehrenreich Barbara Smile or Die: How Positive Thinking Fooled America and The World. London: Granta, 2009.

[R28] Epstein Julia L “Writing the Unspeakable: Fanny Burney's Mastectomy and the Fictive Body.” Representations 16, no. 1 (1986): 131–66. 10.1525/rep.1986.16.1.99p0163m 11618034

[R31] Fearon Henry A Treatise on Cancers, 3rd ed. London: Printed for J. Johnson, 1790.

[R33] Gale Theophilus The Anatomie of Infidelitie, or, An Explanation of the Nature, Causes, Aggravations and Punishment of Unbelief. London: Printed by J. D. for Jonathan Robinson, 1672.

[R34] Guy Richard Practical Observations on Cancers and Disorders of the Breast. London: Printed for W. Owen, 1762.

[R35] Gwinnett Richard Pylades and Corinna: or, Memoirs of the Lives, Amours, and Writings of Richard Gwinnett Esq, 2 Vols London, 1731.

[R36] Hartung Heike “Doleful Ditties’ and Stories of Survival—Narrative Approaches to Breast Cancer in Frances Burney, Maria Edgeworth and Susan Sontag.” Gender Forum 19 (2007): 45–65.

[R12] Hayward Thomas The British Muse, or, a Collection of Thoughts Moral, Natural, and Sublime, of Our English Poets: Who Flourished in the Sixteenth and Seventeenth Centuries, 3 vols London: Printed for F. Cogan, 1738.

[R37] Haywood Eliza The Perplex’d Dutchess: or, Treachery Rewarded. Being Some Memoirs of the Court of Malfy. Dublin: Printed by S. Powell, 1727.

[R39] Hochschild Arlie Russell The Managed Heart: Commercialization of Human Feeling. Berkeley: University of California Press, 1983.

[R41] Kaartinen Marjo Breast Cancer in the Eighteenth Century. London: Pickering and Chatto, 2013.

[R42] King Samantha Pink Ribbons Inc.: Breast Cancer and the Politics of Philanthropy. Minneapolis: University of Minnesota Press, 2006.

[R43] Lavater Johann Casparr Aphorisms on Man: Translated from the Original Manuscript of the Rev. London: Printed for J. Johnson, 1788.

[R44] Lawlor Clark Consumption and Literature: The Making of the Romantic Disease. Basingstoke: Palgrave, 2006.

[R45] Le Prince de Beaumont Jeanne-Marie The Young Ladies Magazine, or, Dialogues Between a Discreet Governess and Several Young Ladies of the First Rank Under Her Education, 4 Vols London: Printed for J. Nourse, 1760.

[R46] Marvell Andrew “Last Instructions to a Painter” Poems on Affairs of State, 7 Vols, edited by Lord GdeF, 99–139. New Haven: Yale University Press, 1963–1975.

[R47] Meek Heather “Frances Burney's Mastectomy Narrative and Discourses of Breast Cancer in the Long Eighteenth Century.” Literature and Medicine 35, no. 1 (2017): 27–45. 10.1353/lm.2017.0001 28529229

[R48] Montagu Walter Miscellanea Spiritualia: or, Devout Essaies. London: Printed for William Lee, Daniel Pakeman, and Gabriel Bedell, 1648.

[R50] Norford William An Essay on the General Method of Treating Cancerous Tumors. London: Printed for J. Noon, 1753.

[R51] Ovid Ovid's Metamorphoses in Fifteen Books. Translated by the Most Eminent Hands. London: Printed for Jacob Tonson, 1717.

[R52] Pindar Peter, (pseud. John Wolcot) Subjects for Painters. London: Printed for G. Kearsley, 1789.

[R53] Piozzi Hester Lynch Anecdotes of the Late Samuel Johnson. London: Printed for T. Cadell, 1786.

[R54] Pope Alexander The Works of Alexander Pope Esq. in Nine Volumes Complete. With his last Corrections, Additions, and Improvements, 9 Vols London: Printed for J. and P. Knapton, 1751.

[R55] Pritchard Rhys The Welshman's Candle: or the Divine Poems of Mr. Rees Prichard, Sometime Vicar of Landovery, in Carmarthenshire. Carmarthen: Printed for the Translator by J. Ross, 1771.

[R57] Reddy William M The Navigation of Feeling: A Framework for the History of Emotions. Cambridge: Cambridge University Press, 2001.

[R58] Rosenwein Barbara H Generations of Feeling: A History of Emotions, 600-1700. Cambridge: Cambridge University Press, 2016.

[R59] Rowe Nicholas The Royal Convert. London: Printed for Jacob Tonson, 1708.

[R40] Rowley J. William A Practical Treatise on the Breasts of Women. London: Printed for F. Newbery, 1772.

[R61] Sharp Samuel A Treatise on the Operations of Surgery. London: Printed by J. Watts, 1739.

[R62] Skuse Alanna Constructions of Cancer in Early Modern England: Ravenous Natures. Houndmills: Palgrave, 2015. 31577403

[R63] Smith Adam The Theory of Moral Sentiments, edited by Raphael D. D and Macfie A. L Indianapolis: Liberty Fund, 1984.

[R64] Smyth Maria The Woman of Letters; or the History of Miss Fanny Belton, 2 Vols London: Printed for Francis Noble, 1783.

[R65] Sontag Susan Illness as Metaphor and AIDS and Its Metaphors. New York: Anchor Books, 1990.

[R72] Störck Vonn, and Freiher Anton An Essay on the Medicinal Nature of Hemlock. Edinburgh: Printed by A. Donaldson and J. Reid, 1762.

[R67] Todd Janet Sensibility: An Introduction. London: Methuen, 1986.

[R68] Todd Janet “The Sentimental Novel” The Encyclopedia of the Novel, edited by Schellinger P Chicago: Fitzroy Dearborn, 1988 Literature Online.

[R69] Torney Kay “Fanny Burney’s Mastectomy. Meridian: The La Trobe University.” English Review 10 (1991): 79–85.

[R71] Trigg Stephanie Emotional Histories—Beyond the Personalization of the past and the abstraction of affect theory, 3–15: Spring, 2014.

[R73] Waters Nicholas A System of Surgery, Extracted from the Works of Benjamin Bell of Edinburgh, edited by Waters N. B Philadelphia: T. Dobson, 1791.

[R75] Whytt Robert Observations on the Nature, Causes, and Cure of those Disorders Which Have Been Commonly Called Nervous Hypochondriac. Edinburgh, 1765.

[R76] Wiltshire John “Fanny Burney’s face, Madam D’Arblay’s Veil” Literature and Medicine during the Eighteenth Century, edited by Roberts M.M. and Porter R., 245–65. London: Routledge, 1993.

